# Supervised administration of primaquine may enhance adherence to radical cure for *P. vivax* malaria in India

**DOI:** 10.1016/j.lansea.2023.100199

**Published:** 2023-04-15

**Authors:** Manju Rahi, Preeti Rana Sirohi, Amit Sharma

**Affiliations:** a Indian Council of Medical Research, New Delhi, India; b Academy of Scientific and Innovative Research (AcSIR), Ghaziabad, Uttar Pradesh, India; c International Centre for Genetic Engineering and Biotechnology, New Delhi, India

**Keywords:** Malaria, Plasmodium vivax, Primaquine, Directly observed therapy

## Abstract

The *Plasmodium vivax* lifecycle encompasses a dormant liver-stage known as 'hypnozoite’ which serves as silent reservoirs of malaria, reactivation of which results in recurring episodes of relapse with varying periodicity. This contributes to continuous transmission of malaria unamenable to control methods. The prevention of relapse requires a “radical cure” by a hypnozoitcidal drug. Primaquine (PQ) has been the recommended radical cure for this malaria. However, adherence to 14 days PQ treatment remains poor. India accounts for majority of *P. vivax* burden globally. However, PQ administration is not supervised in the current national programme. Supervised administration of drugs ensures compliance and improves drug regime success rate. Trials across different countries have established the effectiveness of directly observed therapy (DOT) for prevention of relapses. As India aims to eliminate malaria by 2030, it is prudent to consider DOT to ensure complete treatment of the malaria affected populations. Therefore, we recommend that the Indian malaria control programme may consider DOT of primaquine for treatment of vivax malaria. The supervised administration would entail additional direct and indirect costs but will ensure complete treatment and hence minimize the probability of relapses. This will help the country in achieving the goal of malaria elimination.

## Introduction

The world continues to suffer from malaria as 241 million estimated global cases and 627,000 deaths occurred in the year 2020 as per World Malaria Report 2021.^1^ Out of these total estimated cases, 2% (4.5 million) are due to *Plasmodium vivax.* Although in India, the reported proportion of *P. vivax* and *Plasmodium falciparum* malaria has been equal in the last two decades, but vivax malaria has shown fluctuating trends in the recent years being 38% in 2017, 52% in 2018, 54% in 2019, 36% in 2020 and 37% in 2021.^2^

Human malaria is caused majorly by *P. falciparum* and *P. vivax* species*. P. falciparum* is known to cause higher mortality than *P. vivax* malaria and hence has received more attention and resources globally.^3^^,^^4^ The distinctive feature of *P. vivax* malaria is the liver-stage hypnozoites that have an ability to lie dormant in liver for several months to years and can get reactivated to cause fresh blood stage infection.^5^ Typically, the parasite strains found in the tropical regions mostly exhibit shorter relapse intervals viz. between 3 and 8 weeks, while those found in the temperate and sub-tropical zones exhibit relatively longer dormant period (8–10 months or longer) between the occurrence of primary infection and its relapse.^6^ The relapse patterns vary from one region to another. However, the explicit mechanisms of trigger of relapses and the phenotypic variations remain obscure. The periodic relapses result in early disease tolerance, attributing to high threshold for fever, and sometimes leading to the occurrence of asymptomatic infections.^7^

The clinical manifestations of vivax malaria and the pathobiology behind it presents a wide spectrum of the disease. *P. vivax* has been considered a benign infection. However, a recent systematic review revealed that pooled proportion of severe vivax malaria was 29.7% with symptoms including jaundice, severe anaemia, multi-organ failure and thrombocytopenia.^8^ In a study carried out in a referral hospital of Delhi, North India, out of 177 vivax malaria patients, ∼33% had manifestations of severe malaria.^9^ *P. vivax* malaria is now considered as a pertinent risk factor for severe anaemia and chronic malnutrition particularly in young children of endemic areas.^10^, ^11^, ^12^, ^13^ Malaria contracted during pregnancy potentially leads to maternal anaemia, low birth weight new-borns, premature deliveries and spontaneous abortions. In view of above short and long term debilitating effects of *P. vivax* malaria, it is imperative that the infection is completely treated including extermination of hypnozoites.

As per Indian national treatment guidelines, the drug treatment of *P. vivax* malaria comprises chloroquine (CQ) at 25 mg/kg dosage for three-days followed by 0.25 mg/kg primaquine for 14 days. Primaquine is available as tablets in the strengths of 2.5 mg, 7.5 mg and 15 mg. Though primaquine dose is according to the weight, but for simplicity sake, an age-wise chart is provided for administration of the drug in the national programme. Table 1 represent the dosing for primaquine across ages as per guidelines for diagnosis and treatment of malaria in India (2014).^14^ Studies in Indian and international settings reveal efficacy of chloroquine-primaquine regimen in uncomplicated *P. vivax* malaria.^15^, ^16^, ^17^, ^18^, ^19^ Therefore, it is worthwhile to continue with the existing drugs at optimum compliance rate.

**Table 1:** Dosing table for primaquine across ages as per guidelines for diagnosis and treatment of malaria in India (2014).

Age (years)	Daily dosage (in mg base)
<1	Nil
1–4	2.5
5–8	5.0
9–14	10.0
>15	15.0

Table 2 summarizes the recurrence rates achieved with the 14-day 0.25 mg/kg drug regimen as per various Indian studies with varying duration of follow-ups.

**Table 2:** Recurrence rates achieved with 14-day PQ drug regimen in different Indian.

Study & Year	Place of Study	Number of participants	Duration of follow-up (months)	Rate of recurrence
Gogtay et al., 1999^20^	Mumbai	242	6	0%
Rajgor et al., 2003^21^	Mumbai	398	7	13.6%
Kim et al., 2012^22^	Kolkata	42	6	8.1%
Swagata et al., 2013^23^	Kolkata	250	42-day	0%
Saravu et al., 2018^24^	South India	50	6	8%
Gandrala et al., 2022^25^	Manipal, Karnataka	294	23	8.2%

## ‘Radical cure’ as an anti-relapse treatment

In addition to the phenomenon of reactivation of hypnozoites combined with the poor compliance rate of primaquine treatment, there is an increased risk of *P. vivax* parasitaemia post falciparum malaria treatment, which further complicates the situation in co-endemic areas as highlighted through meta-analysis.^26^ The recommended ‘radical cure’ contains a schizonticide chloroquine for 3-days and the hypnozoicitidal drug primaquine for 14-days. A randomized clinical trial published recently showed additional benefit of radical cure as an adjunct treatment in P. *falciparum* infections in preventing triggering of vivax relapses.^27^ However, in case of chloroquine resistance, Artimisinin-based Combination therapy along with primaquine is recommended.

An alternate to primaquine drug therapy is tafenoquine, which is also an 8-aminoquinoline anti-hypnozoite drug but is a single dose treatment. Primaquine till long was the only 8-aminoquinoline approved for providing a radical cure. The longer-acting anti-hypnozoite, tafenoquine (TQ) has been recently registered with the Food and Drug Administration (U.S.) and Therapeutic Goods Administration (Australia) for resolution of *P. vivax* malaria. It is administered as a single 300 mg dose in combination with a schizonticide, making it more advantageous over primaquine in terms of convenience and hence possibly improved adherence.^28^ Owing to the fact that the complexity of regimen inversely affects the rate of compliance, therefore, TQ coupled with chloroquine would appear to be an excellent option as a single-dose radical cure of *P vivax*. However, the prolonged exposure caused by its long half-life can result in haemolysis in G6PD deficient patients. Thus, the use of tafenoquine is restricted to patients with more than 70% G6PD enzyme activity. Diagnosis of G6PD deficiency at this more stringent threshold requires a quantitative assay, which adds cost and complexity to large-scale roll-out. At the same time, a companion point of care diagnostic device for quantitative assessment of G6PD enzyme activity could make tafenoquine a more viable option for treatment of vivax malaria after ruling out G6PD deficiency. Contrarily, primaquine is rapidly eliminated and thus treatment can be stopped at the first signs of haemolysis. Therefore, primaquine can generally be prescribed safely in people with G6PD enzyme activity greater than 30%. For this reason, the direct supervision of safe and effective radical cure with primaquine is more practical. Moreover, tafenoquine is currently not approved in India.

## Supervised treatment for improved adherence rates: Indian and international scenario

The outcomes of numerous primaquine efficacy studies wherein patients are either unsupervised, or partially supervised, have substantiated the fact that the efficacy of primquine drug is 3–4 times higher in the group which completed the course as compared to the others.^27^^,^^29^, ^30^, ^31^ Adherence is improved when patients are tested, have a definite diagnosis of the health condition, and are directly observed for drug intake. Some of the measures of assessing adherence include self-reporting, pill count and biological assays.

Self-reporting of intake is used widely since it is relatively easy and cheap to implement. However, it is subjective, highly dependent on how questions are asked, prone to recall bias and social desirability bias. Likewise, pill counts are also simple to implement using either manual counting or an electronic pill cap. Nonetheless, these methods are highly unreliable since they tend to overestimate adherence as the patients may be influenced by recall or reporting bias.^32^ Biological assays include detection of metabolized PQ and quantification of methaemoglobin (Met-Hb) concentrations. PQ is metabolized rapidly with a half-life of less than 6 h, and so may be undetected within 24 h of administration. Whereas, its metabolite, carboxy-PQ, is slowly eliminated and accumulates over the course of a 14-day course and may therefore be a more useful measure of adherence. Similarly, quantification of methaemoglobin (Met-Hb) concentrations is another potential proxy measure of adherence. Met-Hb concentrations are elevated following PQ administration which can be measured non-invasively using a finger probe to quantify arterial oxygen-Hb saturation. However, validation of these biological approaches is needed and clinical studies are underway to explore these.^33^ Nonetheless, the method of dosing supervision has been shown to improve adherence, as corroborated through a study by Poespoprodjo and others, wherein the supervised group was monitored for treatment intake on every second day.^27^

There are no reliable markers to differentiate relapse from recrudescence or reinfection for *P. vivax*. In addition, no robust systematic study has been conducted in India to assess compliance rates of primaquine for 14 days. In the absence of above data, it is difficult to comment upon efficacy of primaquine in prevention of relapses in India. However, follow up studies over time have shown a variable recurrence rates of up to 44% when followed for up for 5 years.^20^^,^^21^^,^^34^^,^^35^ It is commonly understood, though there are not direct studies, that the compliance to radical cure of 14 days would be poor in India as in any other low and middle income country and a significant proportion of malaria patients may not be adhering to the stipulated duration.^31^ The inadequacy in adherence to primaquine treatment deprives the patients of full benefit of the hypnozoiticidal effect of primaquine and renders them susceptible to relapses as a result of incomplete treatment of vivax malaria.

Private health care sector plays a major role in providing healthcare to the masses in India. Formal and informal private practitioners co-exist in the healthcare sector in India. When access to trained service providers is limited, due to cost or location, untrained or unqualified private health care providers are approached who may not be following the national guidelines as shown in a review by May Sudhinaraset et al. that revealed low adherence to national guidelines by the untrained and unqualified healthcare professionals.^36^ Urban malaria, which is mostly vivax malaria, is a significant but under-recognized problem in India. Therefore, it is important that private health care sector is brought on board and fully engaged with in order to improve the adherence to national guidelines of radical cure by primaquine.

## Directly observed therapy of *P. vivax* malaria

Under the Indian national treatment guidelines (2014), it is recommended to administer primaquine under supervision with caution to the patients on signs of hemolysis like dark colored urine, bluish discoloration of lips, yellow conjunctiva etc. However, at present the supervision is not direct and likely that patients are just provided the primaquine tablets and not truly monitored whether the patients are consuming tablets or not. The symptoms of the patients settle down in the first 3–4 days of illness and they may not feel encouraged to complete the treatment course. In all likelihood, the patients' adherence to 14 days of radical cure is staggered and variable over 2 weeks’ time and a small fraction of patients only may be completing the 14 days regime.

In order to ensure compliance, supervised administration of drugs (such as in tuberculosis in India) is a well-known strategy to improve the success rate of completion of drug regime.^37^^,^^38^ Several trials have been conducted across countries like Brazil, Indonesia, Thailand and Pakistan where supervised primaquine administration has yielded lower incidence of recurrence of malaria in the supervised group versus unsupervised group self-administered therapy.^27^^,^^39^, ^40^, ^41^ The studies are summarized in Table 3.

**Table 3:** Studies showing impact of supervised primaquine radical treatment in preventing the re-occurrence of vivax malaria in different settings.

Year of publication	Country	Study design	Study period	Method of supervision	Results
2022^27^	Papua, Indonesia	Cluster-randomized controlled trial	2016–2018	Alternate day home visits and provision of PQ tablets for that day and next day till the completion	The incidence risk and incidence rate of *P. vivax* recurrence was higher in the unsupervised group versus the supervised. These findings were found for either *P. falciparum* or *P. vivax* malaria patients.
2021^40^	Brazil	Randomized controlled trial	2019–2020	Daily supervision by home visits and administration of PQ	Significant difference and a lower risk of recurrence in the supervised group as compared to unsupervised upto 180 days of follow up
2011^31^	Thailand (Thai-Myanmar border)	Randomized controlled trial	2005 to 2006	Daily supervision by home visits and administration of PQ	There were no cases of reappearance of *P.vivax* in directly observed therapy group as compared to 5 cases in self-administered therapy group during the follow up period upto 90 days
2004^39^	Pakistan	Cluster randomized control trial	2000–2001	Daily supervision of administration of PQ	There was no significant difference in the supervised and unsupervised group but the placebo group had higher reappearance rate.
2001^42^	Brazil	Prospective open trial without a control group	1997–1998	Supervised administration of PQ through home/hospital visit or follow up with patient on daily basis	Despite supervision, the relapse rate was 2.4 relapses per 100 persons-months in the follow up period of 180 days

In a randomized study from the Thai-Myanmar border during 2007–2009, the *P. vivax* malaria patients were given primaquine either through self-administered therapy (SAT) or by directly observed therapy (DOT) approach. After a follow-up period of 90-days, it was revealed that those who received DOT were ∼6 times less likely to experience the reappearance of *P. vivax* malaria as compared to SAT patients.^31^ This indicated that the re-emergence of *P. vivax* malaria among SAT patients is attributed to non-adherence of the patients to the specified primaquine dosage. Similarly, another study was conducted in 2005–2006 at Ratchaburi (Thailand) along the Thai-Myanmar border for evaluating the recurrence of *P. vivax* malaria infection with regard to the drug compliance. A total of 92 vivax malaria positive patients enrolled for the study and were categorized into 2 separate groups. A standard dose of chloroquine for 3 days was administered in both the groups which was followed by primaquine treatement for 14 days. Patients of the control group were given the medication with necessary instructions to take as SAT, whereas those of the experimental group received a full course of treatment using daily DOT. All of the study subjects were followed up for 3 months. The study outcome revealed that of the 46 patients in SAT group, 5 showed malaria recurrence, whereas no recurrence was observed among patients of the DOT group. Moreover, significant differences were observed between the SAT and DOT groups during survival analysis (p < 0.05).^41^ These studies suggested that when primaquine was administered to patients by DOT, it enhanced overall effectiveness.

Consequently, it is vital that India consider DOT to ensure complete treatment of the malaria affected populations. Additionally, the supervision will also help in monitoring the signs and symptoms of haemolysis such as dark coloured urine as a result of primaquine administration in G6PD deficient individuals. Under the National Health Mission, the grassroot level workers like Accredited Social Health Activists (ASHA), health care workers like Auxillary Nurse Midwife and Multi Purpose Worker are the backbone of any public health programme. ASHAs make several rounds to the households for dispensing of healthcare services ranging from immunization to family welfare and control of vector borne diseases including malaria. They can be trained in carrying out the supervision of primaquine treatment for vivax malaria at the household level. The most optimal strategy can be worked out like alternate day supervision, pill count, examination of blister pack and others.

Incentivization of the healthcare volunteers and patients on completing the treatment, as for post kala azar dermal leishmaniasis and visceral leishmaniasis, can assist in achieving this aim.^43^ Mechanisms need to be developed in order to encourage private health care providers to dispense supervised treatment. Fully realizing several challenges that DOT will pose such as increase in the workload on healthcare workers, additional financial burden on the health system and enhanced training needs, we see it as a vital step towards vivax malaria elimination in India.

## Limitations of directly observed therapy

PQ administration through direct supervision will include monitoring of drug intake by healthcare worker. This will incur additional direct and indirect costs to the national programme by adding the component of DOT in terms of training, resources, travel time and diversion from other activities. However, ensuring compliance will circumvent the problem of malaria relapses which would greatly contribute to the efforts made towards achieving malaria elimination goal.

Table 4 outlines the advantages and disadvantages of SAT and DOT as a means of PQ administration.

**Table 4:** Comparative analysis of Self Administered Therapy versus Directly Observed Therapy for PQ administration.

Features	PQ Self Administered Therapy	PQ Directly Observed Therapy
Administration	Patient takes the drug on his/her own	Drug intake monitored by healthcare worker
Periodicity of supervision	None	Daily or alternate days
Mechanism of supervision	Supervision is none but instructions given to patients	• Visit by healthcare workers to home or patients called to healthcare facility. • Digital tools via phone calls/SMS alerts or social media
Advantages	• No additional effort by HCW • No additional cost to the health system • Entire course of treatment given in one go especially advantageous for mobile population	• Ensured consumption of drug by the patient and minimize the risk of relapse. • Any adverse reaction of PQ can be reported by patient/enquired by HCW at regular basis and hence can be managed better • Enhances patient's confidence in health care system and improves access to timely healthcare • More opportunities for patients via multiple contacts to report further febrile episodes (possibly malaria).
Disadvantages	• No assurance of complete compliance • Adverse reactions may go undetected, unreported and hence unmanaged • Increased chances of relapses if adherence by patient is incomplete • Wastage of medicines if patient just collects and is unwilling to complete the treatment	• Additional cost and effort by healthcare system • Time consumed in supervision needs to be diverted from her schedule of other healthcare activities.
Cost	0.15–0.60 USD per course	Cost of medicine + additional human and logistical costs of supervision

## Conclusion

In view of the evidence generated by the studies enumerated above where higher compliance rates and reduced incidence rate of vivax malaria in the supervised group were observed, we strongly recommend that the national malaria control programme can adopt the strategy of directly supervising the primaquine intake for 14 days. This will improve compliance and hence reduce the risk of relapses of vivax malaria.

## Contributors

MR and AS: Conceptualization, interpretation, supervision, writing, review and editing; PRS: Data curation, writing, review & editing; All authors read and approved the paper.

## Declaration of interests

The authors declare no conflict of interests.
